# Physical Activity Improves Cognition and Activities of Daily Living in Adults with Alzheimer’s Disease: A Systematic Review and Meta-Analysis of Randomized Controlled Trials

**DOI:** 10.3390/ijerph19031216

**Published:** 2022-01-22

**Authors:** Shengwen Zhou, Sitong Chen, Xiaolei Liu, Yanjie Zhang, Mengxian Zhao, Wenjiao Li

**Affiliations:** 1 Department of Chinese Martial Arts, College of Sport Science, Hunan University of Science and Engineering, Yongzhou 425100, China; zhoushengwen81@163.com; 2 Institute for Health and Sport, Victoria University, Melbourne 8001, Australia; sitongchen@szu.edu.cn; 3 Chinese Traditional Regimen Exercise Intervention Research Center, Beijing Sport University, Beijing 100084, China; liuxiaolei99@hotmail.com; 4 Health and Exercise Science Laboratory, Institute of Sports Science, Seoul National University, Seoul 08826, Korea; zhangyanjie@cuhk.edu.cn; 5 Physical Education Unit, School of Humanities and Social Science, The Chinese University of Hong Kong—Shenzhen, Shenzhen 518172, China; 6 School of Physical Education, Shenzhen University, Shenzhen 518060, China; helenzhao2020@126.com

**Keywords:** physical exercise, cognitive function, activities of daily living, Alzheimer’s disease

## Abstract

Objective: The purpose of this meta-analysis was to examine the effects of physical activity (PA) on cognition and activities of daily living in adults with Alzheimer’s Disease (AD). Methods: Six electronic databases (MEDLINE, CINAHL, PsycArticles, SPORTDiscus, EMBASE and CNKI) were used to search for potential studies from inception until October 2021. Randomized controlled trials (RCTs) investigating the effect of physical activity (PA) on cognition and activities of daily living in AD patients compared to a control condition were included. The effect sizes were synthesized using a random effects model with a 95% confidence interval (CI). Results: Sixteen articles including 945 participants (aged 70 to 88 years, 34.6% male) met the inclusion criteria. The pooled effect sizes demonstrated that PA intervention was associated with significant improvements in global cognition (Standard Mean Difference (SMD) = 0.41, 95% CI [0.24, 0.58], *p* < 0.01) and activities of daily living (SMD = 0.56, 95% CI [0.32, 0.79], *p* < 0.01) in AD patients. Subgroup analyses suggested that PA for 3–4 times per week for 30–45 min for more than 12 weeks had a relatively strong effect on improving global cognition in AD patients. The sensitivity analysis showed robust results. Conclusions: The findings from the current meta-analysis suggested that AD patients can improve their global cognition and Activities of Daily Living (ADL) through engaging in aerobic and mixed exercise (aerobic and anaerobic exercise).

## 1. Introduction

Alzheimer’s disease (AD) is the most common type of dementia, accounting for 60–80% of the confirmed cases. It is a chronically non-communicable, irreversible, neurodegenerative disorder, primarily appearing in the population aged 65 years and older [1,2]. The causes of AD are multifactorial, including gene mutation, age, having less physical activity, lower education levels, and cardiovascular disease and complications [1]. Individuals diagnosed with AD have different symptoms, including memory impairment, apathy, disorientation, behavior changes, and even difficulty walking and speaking. Moreover, AD can result in a poor quality of life and a loss of independence [1]. According to the World Health Organization (WHO), approximately 50 million people have been diagnosed with dementia worldwide, and the recently estimated number will be increased to 82 million in 2030 and 152 million in 2050 [3]. 

Currently, faced with the above problem, it is necessary to adopt more effective methods to improve AD patients’ quality of life and health outcomes. Nevertheless, there are no specific and compelling drugs for treating AD, so researchers have a strong awareness of the shift from pharmacotherapy to physical activity (PA) and expect to find a cure approach for AD. A growing body of studies has suggested that PA has been considered a non-pharmacological therapy associated with improved mental health (e.g., depression and anxiety) and cardiovascular fitness [4,5,6]. Moreover, existing evidence has shown a positive relationship between more PA and cognitive performance in adults [7,8,9]. Therefore, as a complementary therapy, the PA plays a vital role in improving physical fitness, mental health, and even cognition in different populations. Specific to unhealthy aging, the existing reviews have not presented a comprehensive understanding of the effects of PA on cognition in AD patients. Previous studies have suggested that older adults should engage in PA to slow down cognitive impairment, especially dementia patients [10,11,12,13]. For instance, a review study with 26 longitudinal studies conducted by Blondell et al. found that a higher level of physical activity (i.e., aerobic and anaerobic exercise) was associated with a reduced risk of cognitive decline in dementia [14]. However, positive results are not always observed in systematic reviews. A recent systematic review conducted by Borges-Machado et al. shows uncertain effects of exercise on cognition in adults with dementia [15]. Similarly, Demurtas and colleagues could not be certain that PA improved cognition in dementia patients [16]. To support this point, a recent meta-analysis study including 11 studies showed no significant improvements in a Mini–Mental State Examination (MMSE) test [17]. 

In addition to cognitive decline, dementia patients also have a significant impairment in physical function, especially for Activities of Daily Living (ADL). The level of ADL capacity directly affects the quality of life and the burden cost of care in patients [18,19,20]. Many studies have documented that PA is a crucial way to improve muscle strength, muscle mass, balance, ADL dependence [2,21,22], and therefore their quality of life [23]. For example, exercise (e.g., aerobic and resistance exercise) helped improve the daily life capacity in individuals with dementia [24]. A recent study found that dementia patients showed improvements in ADL performance [15] and quality of life [25] after doing long-term PA. However, most systematic review studies have investigated ADL in adults with dementia, and little research has been done on the effects of exercise and ADL in people with AD. Meanwhile, the existing evidence from different studies is also inconsistent [10,26]. 

Although many systematic review studies have examined the effects of exercise on cognition and physical performance in adults with AD, they have commonly shown inconclusive results due to inconsistent inclusion criteria, considerable heterogeneity (*I*^2^ ≈ 80%) across studies, a small number of included studies, and different types of dementia populations (e.g., Lewy body dementia and vascular dementia) [16,17,27]. They have considered the limitations of previous studies and the fact that a growing body of new RCT studies published in Chinese and English language is investigating the effect of exercise on cognition and ADL in populations with AD. Promising and robust evidence is expected to confirm the effects of PA on promoting cognitive function and ADL in individuals with AD. Therefore, there is a need to conduct an updated meta-analysis (1) to investigate the effects of PA on global cognition and ADL in adults with sole AD; and (2) to evaluate the impacts of the attributes of PA on cognition using moderator analysis based on the exercise dose (frequency, type, time, and duration), the stage of AD, and the study design.

## 2. Methods

### 2.1. Search Strategy 

Articles were retrieved from six electronic databases (MEDLINE, CINAHL, PsycArticles, SPORTDiscus, EMBASE and CNKI) from their inception until October 2021. In each database, we combined the following groups of terms for potential studies: (1) “physical activity” OR “physical training” OR “exercise” OR “aerobic exercise” OR “cycling” OR “walking” OR “resistance training” OR “strength exercise”; (2) “Alzheimer’s disease” OR “Alzheimer*” OR “AD”; (3) “randomized controlled trials”. Additional articles were manually found by studying the reference lists of the included studies. PRISMA (Preferred Repointing Items for Systematic Reviews and Meta-Analyses) was used in the current study [28].

### 2.2. Inclusion and Exclusion Criteria

The included articles met the following criteria: (i) participants had a clinical diagnosis of AD based on the National Institute of Neurological and Communicative Disorders and Stroke and the Alzheimer’s Disease and Related Disorders Association (NINCDS-ADRDA), as well as the Diagnostic and Statistical Manual of Mental Health Disorders (DSM-IV); (ii) the interventions involved one or more similar PA therapies (e.g., walking, or walking and stretching) in the experimental group and either a placebo-control condition or a control condition, without using any other exercise interventions in the control group; (iii) the study design consisted of randomized controlled trials (RCTs); (iv) at least one outcome measurement (i.e., ADL and global cognition, e.g., (Mini-Mental State Examination (MMSE), the Cognitive section of the Alzheimer’s Disease Assessment Scale (ADAS-Cog), and the Rapid Evaluation of Cognitive Function (ERFC))) was reported; (v) studies were published in English or Chinese. Studies were excluded when (i) they were animal or in vitro studies, abstracts, case reports, reviews, or trial protocols; (ii) there were participants with other types of dementia (e.g., vascular dementia) or mild cognitive impairment; (iii) outcome measurements could not be used to calculate the effect size (ES).

### 2.3. Data Extraction and Quality Assessment 

Eligible studies were evaluated by two independent authors, screening the titles, abstracts, and full texts. When disagreement appeared, a third author would be consulted. The following information was extracted from each included article: author, year of publication, characteristics of subjects, sample size, gender, age, intervention protocol (frequency, time, type, duration and intensity), outcome measurements (ADL and global cognition (e.g., MMSE and ADAS-Cog)), and raw data for effect size calculation. 

The eligibility studies’ risk of bias was assessed by two independent reviewers, using the Physiotherapy Evidence Database (PEDro) scale [29], which is a systematic tool to assess the study quality. This scale encompasses 11 items, of which item 1 is not involved in calculating the total scores. The total valid scores range from 0 to 10. The methodological quality classifications are excellent (9–10 points), good (6–8 points), fair (4–5 points) and poor (<4 points) [29]. 

### 2.4. Statistical Analysis

Meta-analysis was performed using the Comprehensive Meta-Analysis software (v2 software, Biostat, Englewood, NJ, USA). The continuous data on cognition and ADL were extracted from the included studies. Standardized mean difference (SMD) was expressed as the effect size (ES) measure by calculating the mean change from pre- to post-intervention in the experimental and control groups, respectively. The standard deviation was calculated using the equation: SD = SE*√n. The ESs were calculated using a random effects model with a 95% confidence interval (CI) to avoid a high risk of false-positive results [30]. The pooled ESs were presented in a forest plot, and a positive ES value reflected a more effective PA. ES was commonly classified into three levels: small (0.2–0.39), moderate (0.4–0.60), and large (≥0.6) [31]. Heterogeneity was examined using the *I*^2^ statistic for each analysis: *I*^2^ < 25% (low heterogeneity), *I*^2^ < 50% (moderate heterogeneity), and *I*^2^ < 70% (high heterogeneity) [32]. Prediction intervals (PIs) were measured to reflect the variation in intervention effects when more than 10 studies were included in the meta-analysis according to the formula: 95% PI = MD ± 2 Tau^2^ [33]. Sensitivity analysis was performed to evaluate the stability of the results by excluding one study each time. Lastly, the funnel plot, Begg’s test, and Egger’s regression test were employed to examine the publication bias. Duvall and Tweedie’s trim and fill analysis was used to recalculate a new ES by adjusting the outlier values. The significance level was set at *p* < 0.05.

In addition, to investigate the confounding factors (study design, the stage of AD, location, and exercise principles), we performed subgroup analyses and meta-regression tests accordingly. In terms of the exercise principle, a prior study indicated that exercise modalities were likely to influence the findings [34]. Based on the included studies, we slightly modified the cut-off of the exercise duration (short-term: ≤12 weeks; medium-term: 13–23 weeks; long-term: ≥24 weeks) and session time (medium: 30–45 min; longer: >46 min) based on the American College of Sports Medicine [35]. With regard to gender, according to the proportion of males and females in original studies, we coded a high male percentage as ≥40% and a high female percentage as >60%.

## 3. Results

### 3.1. Search Results

**Figure 1:**
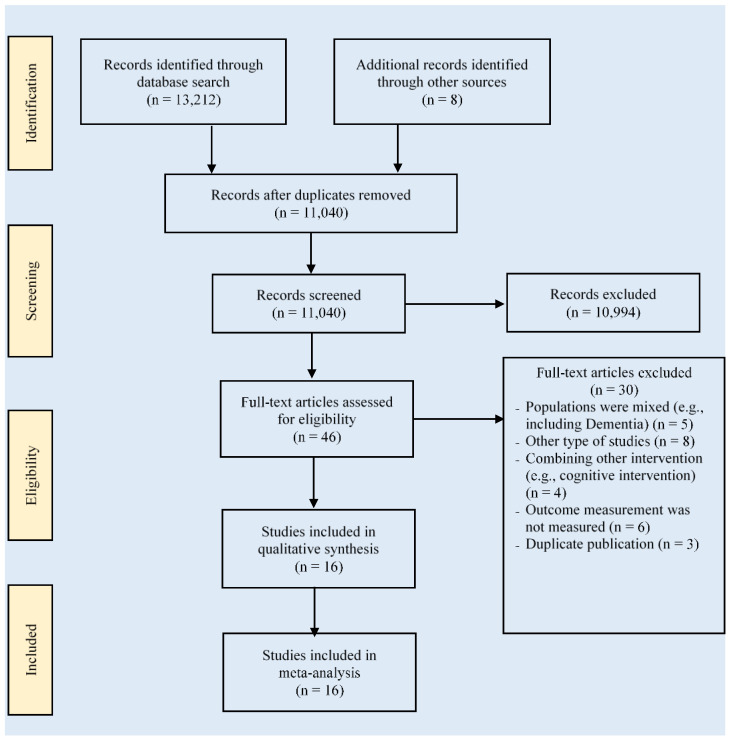
The flowchart of study selection based on PRISMA.

Figure 1 depicts the flowchart of the study selection for this meta-analysis. The initial search record of studies was 13,220. After removing duplicates (n = 2180), 11,040 studies were remaining. Furthermore, 10,994 studies were eliminated after reviewing the titles and abstracts. As a result of the screening process, 30 articles were excluded by assessing the full texts (n = 46), and 16 RCTs [36,37,38,39,40,41,42,43,44,45,46,47,48,49,50,51] met the inclusion criteria in this meta-analysis.

### 3.2. Characteristics of Included Studies

Detailed information of the included studies is shown in Table 1. All studies were published after 2010. The included studies were from Asia, South America, and Europe. The sample size ranged from 20 to 200. A total of 945 participants were diagnosed with mild to severe AD (approximately 34.6% of males). The mean age of the participants ranged from 70 to 88 years. PA protocols were multiple interventions across the included studies. Eight studies used aerobic exercise (e.g., walking, cycling and gymnastics) [43,44,46,47,49,50,51,52], and eight studies used mixed exercises (aerobic exercise and resistance training) [37,38,39,40,41,42,45,48]. Moderate-intensity PA was used in all studies, except one where moderate to high intensity was used [40]. PA duration ranged from three to six months, and the training time for each session was 30 to 90 min, 2–7 times weekly. The participants in the control group of 11 studies [36,37,39,40,41,43,44,45,47,48,50] were mainly given the usual medical treatment (e.g., donepezil). Five studies used social activity [38,42,45,46,49], two studies used cognitive treatment [39,47], and one study used a health education intervention [51]. Regarding outcomes, global cognition (MMSE, ADAS-Cog and ERFC) and ADL (Barthel Index and Alzheimer’s Disease Cooperative Study—Activities of Daily Living Inventory) were usually considered in sixteen [36,37,38,39,40,41,42,43,44,45,46,47,48,49,50,51] and eight studies [36,38,39,41,44,48,49,50], respectively.

According to the Physiotherapy Evidence Database (PEDro) scale, the methodological quality of each study is presented in Table 2. The study quality score fluctuated from six to eight, implying that all studies were considered to be of good methodological quality. Concealed allocation was used in eight studies [37,38,39,40,42,44,49,50]. Only two studies performed a blinding method to measure the outcomes [39,40].

### 3.3. Effects of PA Intervention on Global Cognition

**Figure 2:**
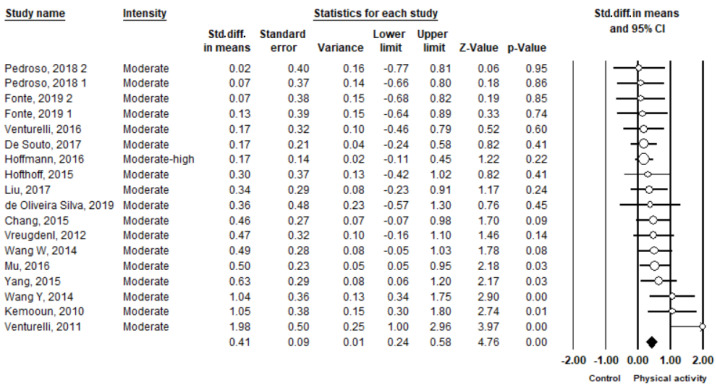
Forest plot showing the effects of physical activity vs. control on global cognition.

**Figure 3:**
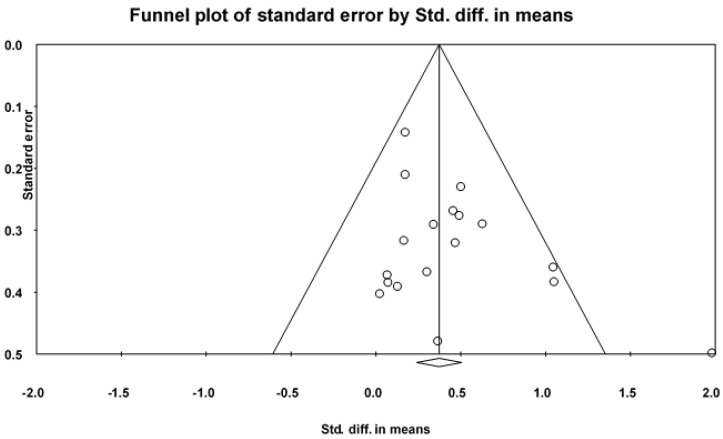
The funnel plot of publication bias for global cognition.

The pooled result from 18 studies showed a significant improvement in favor of PA (SMD = 0.41, 95% CI 0.24 to 0.58, *I*^2^ = 30%, *p* < 0.01; 95% PI = −0.04 to 0.85) (Figure 2, Supplement Figure S1). However, the PI revealed that PA may not elevate global cognition in more than 5% of trial settings. By removing one study at a time, the pooled result of global cognition was stable. A symmetrical funnel plot was shown in Figure 3, indicating no publication bias for global cognition. Begg’s test (Kendall’s tau = 0.07, *p* = 0.74) and Egger’s test (intercept = 1.48, *p* = 0.08) indicated no publication bias. Furthermore, the ES was recalculated using Duvall and Tweedie’s trim and fill procedure, adding two studies on the right sides of the funnel plot, and an adjusted ES was SMD = 0.40 (95% CI 0.28 to 0.60, *p* < 0.05).

**Figure 4:**
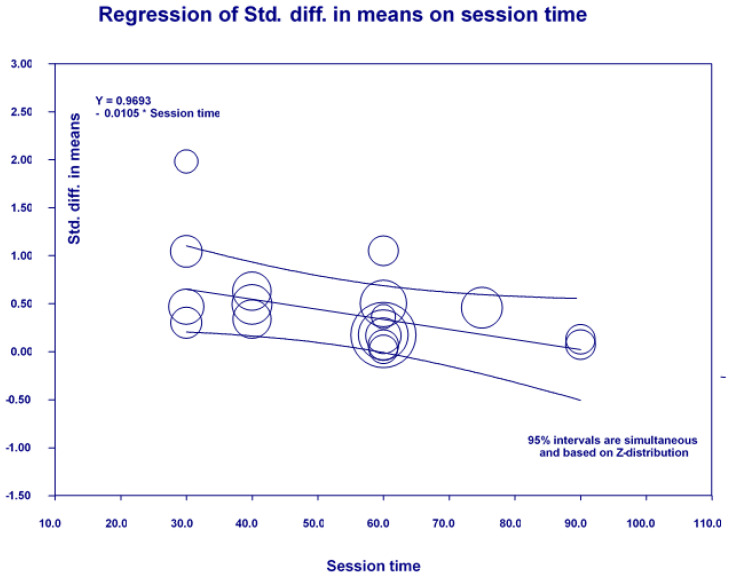
Effect size by exercise session time in meta-regression for global cognition.

Additionally, we conducted subgroup analyses to examine potential factors affecting PA on cognition, shown in Table 3. There were significant differences in how exercise type (*Q*_(1)_ = 4.69, *p* = 0.03), exercise session time (*Q*_(1)_ = 4.41, *p* = 0.04), and AD stage (*Q*_(1)_ = 6.26, *p* = 0.04) affected global cognition. This suggests that aerobic exercise (SMD = 0.60, 95% CI 0.32–0.88) had a greater effect than mixed exercises (SMD = 0.24, 95% CI 0.06–0.41) on global cognition. Moreover, an exercise session time of less than 45 min (SMD = 0.66, 95% CI 0.33–0.99) had a greater effect on global cognition than one of more than 45 min (SMD = 0.27, 95% CI 0.11–0.42). A moderate to severe AD stage (SMD = 0.75, 95% CI 0.03–1.47) had a greater effect on global cognition than a mild to moderate AD stage (SMD = 0.20, 95%CI −0.003–0.39). However, there were no significant differences in exercise duration (*Q*_(1)_ = 0.22, *p* = 0.64), frequency per week (*Q*_(2)_ = 0.79, *p* = 0.67), the stages of AD (*Q*_(2)_ = 5.43, *p* = 0.07), control group type (*Q*_(3)_ = 2.72, *p* = 0.44), or study quality (*Q*_(1)_ = 0.24, *p* = 0.62). In addition, among six continuous predictors (the exercise session time, duration (weeks), frequency per week, total training time during the experiment, participant age, and male percentage) in meta-regression analyses, the session time generated a significant effect on global cognition (β = −0.0105, *p* = 0.03) (see Table 3 and Figure 4).

### 3.4. Effects of PA Intervention on Activities of Daily Living

**Figure 5:**
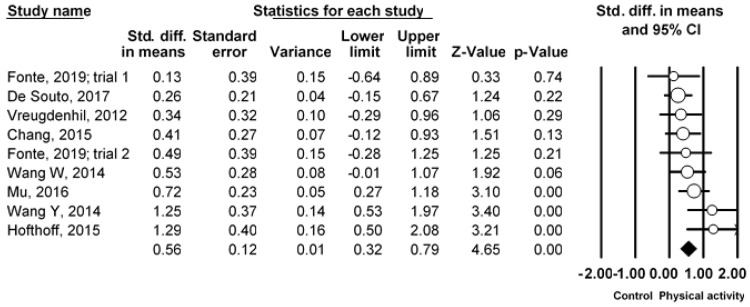
Forest plot showing the effects of physical activity vs. the control on activities of daily living.

**Figure 6:**
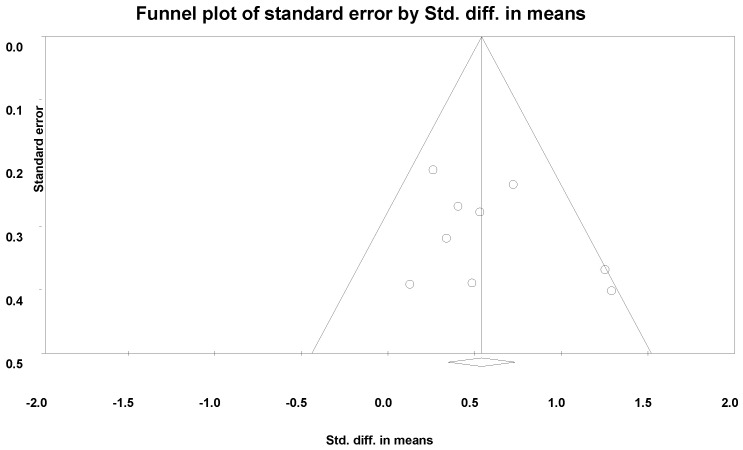
The funnel plot of publication bias for activities of daily living.

Figure 5 shows a forest plot of the meta-analysis. The pooled result from nine studies revealed that the PA intervention had a significant benefit on ADL (SMD = 0.56, 95% CI 0.32 to 0.79, *I*^2^ = 29.86%, *p* < 0.001). By removing one study at a time, the pooled result of ADL was stable. A symmetrical funnel plot was shown in Figure 6, indicating no publication bias for ADL. Begg’s test (*p* = 0.60) and Egger’s test (*p* = 0.29) indicated no publication bias. However, Duvall and Tweedie’s trim and fill procedure revealed that no study was missing after adjusting for outlier values. A new ES was SMD = 0.54 (95% CI 0.32 to 0.79, *p* < 0.05).

## 4. Discussion

This systematic review with meta-analysis examined the effects of PA on adults with AD, as well as the estimated ESs of PA on cognition and ADL. Compared to previous meta-analyses, there were six studies [36,39,43,44,49,50] newly included in this review. Thus, a total of 945 participants (34.6% male) were involved in 16 RCTs. Overall, the methodological quality of the included studies was high. The findings showed that PA significantly benefited global cognition and ADL in adults with AD. 

Although many meta-analyses have indicated the effects of exercise on adults with AD [17,27,53], there are differences in selection criteria between our study and previous studies. Due to the different mechanisms of dementia, abnormal protein deposits may coexist with neuro-vasculature at different stages of dementia and influence brain function [54]. For example, Lewy body dementia is associated with the α-synuclein protein; AD is associated with the *β*–amyloid and *tau*-proteins; vascular dementia is attributed to a lack of blood flow [55]. Therefore, we included participants with only Alzheimer’s type of dementia in this current study. Some meta-analyses intended to target people with AD, but the actual analysis included people with different types of dementia [17,27,53]. Zhu et al. conducted the meta-analysis by including both RCTs and non-RCTs, which directly increased the risk of bias [53]. Moreover, only exercise intervention was considered to meet the inclusion criteria. Some meta-analyses have combined the exercise with other therapies (e.g., psychotherapy) in the experimental group, which influenced the effectiveness of sole exercise [53,56]. Our study, compared to other systematic reviews, avoided the flaws of the above aspects and excluded some original studies. In this context, the findings from previous studies may not necessarily generalize the specific individuals with AD. On the contrary, our meta-analysis presented a low level of heterogeneity (approximately *I*^2^ = 28%, *p* > 0.1) by implementing strict inclusion criteria, showing reliable evidence of the effectiveness of PA on global cognition and ADL in adults with AD. 

With regard to global cognition, all included studies, except for two studies using ADAS-Cog [39] and ERFC [42], examined the effect of PA on global cognition using the MMSE instrument. Both assessment tools have similar components (i.e., memory, attention, orientation, etc.) to evaluate global cognition. Our findings indicated that PA led to a significantly moderate improvement in global cognition (SMD = 0.42) for AD patients compared with the control group. This result was in line with previous meta-analyses, which demonstrated that exercise intervention contributed to an increase in global cognition amongst AD and dementia patients [54,57]. Although our result (SMD = 0.42, *I*^2^ = 30%) is slightly smaller than those of the previous study (SMD = 0.48, *I*^2^ = 85%) [54], the robustness of our result with lower heterogeneity is assured by including new RCT studies published in recent years. However, the PI for global cognition ranged from –0.04 to 0.85, implying that future research projects are likely to report a larger effect in the PA group, although all trials may not show a positive effect. Therefore, the results of this study are useful for researchers or therapists to better target intervention goals and methods, and for patients to know what to expect. 

Additionally, subgroup analysis showed that exercise session time showed a significantly positive correlation with global cognition (*p* = 0.03), and exercise for 30–45 min had a more significant effect than exercise for more than 45 min. However, due to the moderate heterogeneity and a limited number of studies in interventions lasting 30–45 min, the evidence from the subgroup analysis of exercise session time does not very strongly determine whether an exercise session time of 30–45 min is better than one of more than 45 min. Jia et al. found that, in dementia patients, the positive effect on cognition was greater with an exercise session time of up to 30 min compared with one of more than 30 min [58]. Indeed, the included studies show that performing 30–45 min of exercise, compared with more than 45 min, produces better effects on global cognition. According to the current study, we moderately suggested that practicing moderate-intensity PA 3–4 times per week for 30–45 min for more than 12 weeks positively affected global cognition in adults with AD. However, future studies are warranted to verify our recommendation. 

In terms of ADL, the ADCS-ADL is usually used to evaluate primary and operational activities of daily living for individuals with AD. In this current meta-analysis study, six studies directly reported ADL scores using the ADCS-ADL scale [36,38,39,41,49,50], and one study examined the ADL with the Barthel Index [44]. In older adults, impaired cognitive function is associated with difficulties in goal-directed activities (e.g., dressing and household duties) [59]. Reversely, improved cognitive performance leads to an improvement in physical function [59]. After participating in PA, participants showed a significant improvement in their ADL. The results from this current meta-analysis revealed that PA might have a considerable benefit in improving ADL in adults with AD. The calculated ES for ADL was 0.56, which is considered moderate. Because of the limited number of studies, the PI analysis for PA and ADL was not performed. Our finding is in line with previous review studies [53,57], which suggested that PA improved executive function and enhanced the ability of ADL in older adults. Meanwhile, exercise can adequately protect against a decline in ADL by improving skeletal muscle quality [60]. Given the limited number of included studies, subgroup analyses were not conducted to examine the impact of exercise dose on ADL. Further research investigating PA’s effects on improving ADL is needed. It is possible to make firm recommendations on an optimal PA strategy for AD patients.

This meta-analysis was an updated and comprehensive investigation of the beneficial effects of PA on global cognition and ADL. RCTs and sole AD patients were included, which avoids the influence of the bias of non-RCTs or an inconsistency in population. In particular, sensitivity analysis indicated that the findings maintained robust. These findings are vital and timely to help clinicians or caregivers provide exercise guidance to AD patients during physical rehabilitation since PA is a cost-effective intervention for all age groups. It is possible to develop a public health policy to help AD patients prevent cognitive decline. In addition, PA can lead to other health benefits, such as reduced depression and improved quality of life; depression and reduced quality of life are common in AD patients [61,62]. In contrast, the most effective PA program for AD patients is yet to be developed. Long-term participation in PA is more difficult for this population because older adults initiate PA programs and commonly discontinue exercise within half a year [63]. Thus, establishing methods and strategies to encourage AD patients to maintain long-term exercise is needed in future studies. 

Several limitations in this meta-analysis should be considered. First, only articles published in Chinese and English were included, likely resulting in language bias. Second, we only examined global cognition and ADL without analyzing other indicators, such as the subdomains of cognition (attention, processing speed, etc.). Further, the accuracy of these outcomes needs to be examined using other sophisticated instruments (i.e., magnetic resonance imaging) due to the possible bias of self-reported results. 

## 5. Conclusions

To examine the pooled effects of PA on global cognition and ADL in adults with AD, our meta-analysis study included articles with a relatively high methodological quality. The findings from this current study support that both aerobic exercise and mixed exercises benefited global cognition and ADL in patients with AD. Further research with large sample sizes and a high methodological quality is needed to acknowledge these findings.

## Figures and Tables

**Table 1:** Characteristics of the included randomized controlled trials.

Study/Country	Severity of AD Age (Years) Total Sample Size Male (%)	Diagnostic Criteria	Time/Frequency/ Duration/Intensity	Intervention Protocol		Outcomes (Instrument)
				Experiment (Details)	Control	
Chang, 2015 [36] China	Mild to moderate 70.5 n = 57 38.6%	DSM-IV MMSE	60–90 min/3 times/week 16 weeks 50–70% HRmax moderate	aerobic exercise (cycling, walking)	usual medical treatment	global cognition (MMSE) ADL
de Oliveira Silva, 2019 [37] Brazil	Mild 79.4 n = 27 40.7%	DSM-IV	60 min/2 times/week 12 weeks 80% HRmax moderate	mixed exercises (treadmill, weight-lifting, balance exercises)	usual medical treatment	global cognition (MMSE)
de Souto Barreto, 2017 [38] France	Mod to severe 87.5 n = 91 15.4%	DSM-IV MMSE	60 min/2 times/week 24 weeks moderate	mixed exercises (walking, weight-lifting, coordination, balance exercises)	social activity	global cognition (MMSE) ADL
Fonte, 2019 [39] Italy	Mild 79 n = 60 35%	NINCDS-ADRDA	90 min/3 times/week 24 weeks 70% HRmax moderate	mixed exercises (cycling, walking, weight-lifting)	C1: cognitive treatment C2: usual medical treatment	global cognition (ADAS-Cog) ADL
Hoffmann, 2016 [40] Denmark	Mild 70.5 n = 200 56.5%	NINCDS-ADRDA	60 min/3 times/week 16 weeks 70-80% HRmax moderate-high	mixed exercise (ergometer bicycle, cross-trainer, treadmill, strength training)	usual medical treatment	global cognition (MMSE)
Holthoff, 2015 [41] Germany	Mild or moderate 71.5 n = 30 50%	NINCDS-ADRDA	30 min/3 times/week 12 weeks moderate	mixed exercises (movement trainer, resistance training)	usual medical treatment	global cognition (MMSE) ADL
Kemoun, 2010 [42] France	Moderate to severe 82 n = 31 26%	DSM-IV MMSE	60 min/3 times/week 15 weeks moderate	mixed exercises (walking, equilibrium, stamina, ergocycle with the arms and the legs)	social activity	global cognition (ERFC)
Liu, 2017 [43] China	Mild 70.5 n = 48 43.8%	MRI MMSE	40 min/3 times/week 12 weeks moderate	aerobic exercise (aerobic gymnastics: rowing movement, kicking movement)	usual medical treatment	global cognition (MMSE)
Mu, 2016 [44] China	Mild to moderate 73 n = 78 37.2%	NINCDS-ADRDA MMSE	60 min/3 tims/week 16 weeks Moderate	aerobic exercise (brisk walking)	usual medical treatment	global cognition (MMSE) ADL (Barthel Index)
Pedroso, 2018 [45] Brazil	Mild to moderate 78.3 n = 57 24.6%	DSM-IV	60 min/3 times/week 12 weeks 60–75% HRmax Moderate	mixed exercises (walking, stretching, jogging, climbing, and descending stairs)	C1: social activity C2: usual medical treatment	global cognition (MMSE)
Venturelli, 2011 [46] Italy	Severe 84 n = 24 0%	DSM-IV MMSE	>30 min/4 times/week 24 weeks moderate	aerobic exercise (walking)	social activity	global cognition (MMSE)
Venturelli, 2016 [47] Italy	Moderate to severe 84 n = 40 30%	DSM-IV MMSE	60 min/5 times/week 3 months moderate	aerobic exercise (walking)	cognitive treatment	global cognition (MMSE)
Vreugdenhil, 2012 [48] Australia	Mild 74 n = 40 (16/24) 40%	NINCDS-ADRDA	>30 min/7 times/week 4 months	mixed exercises (brisk walking, upper and lower body strength, balance training)	usual medical treatment	global cognition (MMSE) ADL
Wang, W 2014 [49] China	Mild to moderate 70.5 n = 54 38.9%	NINCDS-ADRDA	40 min/3tims/week 6 months Moderate 70% HRmax	aerobic exercise (cycling)	social activity	global cognition (MMSE) ADL
Wang, Y 2014 [50] China	Mild to moderate 70.7 n = 39 33.3%	NINCDS-ADRDA	30 min/3 times/week 12 weeks 50% HR reserve	aerobic exercise (cycling)	usual medical treatment	global cognition (MMSE) ADL
Yang, 2015 [51] China	Mild to moderate 72 n = 50 34%	NINCDS-ADRDA MMSE	40 min/3 times/week 12 weeks 70% HRmax	aerobic exercise (cycling)	health education	global cognition (MMSE)

Note: Total sample size means total number of samples in one experimental study; Male%: the Male percentage in the total sample size; ADAS-Cog: Cognitive section of the Alzheimer’s Disease Assessment Scale; ADL: Alzheimer’s Disease Cooperative Study–Activities of Daily Living Inventory; DSM-IV: Diagnostic and Statistical Manual of Mental Disorders, 4th edition; ERFC: Rapid Evaluation of Cognitive Function; HR: Heart Rate; MMSE: Mini-Mental State Examination; MRI: Magnetic resonance imaging; (NINCDS-ADRDA: National Institute of Neurological and Communication Disorders and Stroke–Alzheimer Disease and Related Disorders Association.

**Table 2:** Methodological quality of the included studies (PEDro analysis).

Study	Score	Methodological Quality	PEDro Item Number										
			1	2	3	4	5	6	7	8	9	10	11
Chang, 2015 [36]	5	Fair	✔	✔		✔				✔		✔	✔
de Oliveira Silva, 2019 [37]	7	Good	✔	✔	✔	✔				✔	✔	✔	✔
de Souto Barreto, 2017 [38]	6	Good	✔	✔	✔	✔				✔		✔	✔
Fonte, 2019 [39]	8	Good	✔	✔	✔	✔			✔	✔	✔	✔	✔
Hoffmann, 2016 [40]	8	Good	✔	✔	✔	✔			✔	✔	✔	✔	✔
Holthoff, 2015 [41]	5	Fair	✔	✔		✔				✔		✔	✔
Kemoun, 2010 [42]	6	Good	✔	✔	✔	✔				✔		✔	✔
Liu, 2017 [43]	6	Good	✔	✔		✔				✔	✔	✔	✔
Mu, 2016 [44]	7	Good	✔	✔	✔	✔				✔	✔	✔	✔
Pedroso, 2018 [45]	5	Fair	✔	✔		✔				✔		✔	✔
Venturelli, 2011 [46]	5	Fair	✔	✔		✔				✔		✔	✔
Venturelli, 2016 [47]	6	Good	✔	✔		✔				✔	✔	✔	✔
Vreugdenhil, 2012 [48]	6	Good	✔	✔		✔				✔	✔	✔	✔
Wang, W 2014 [49]	7	Good	✔	✔	✔	✔				✔	✔	✔	✔
Wang, Y 2014 [50]	7	Good	✔	✔	✔	✔				✔	✔	✔	✔
Yang, 2015 [51]	6	Good	✔	✔		✔				✔	✔	✔	✔

Note: PEDro, Physiotherapy Evidence Database scale; Studies were classified as having excellent (9–10), good (6–8), fair (4–5), or poor (<4). Scale of item score: ✔, present. The PEDro scale involves (1) eligibility criteria; (2) random allocation; (3) concealed allocation; (4) similarity at baseline on key measures; (5) participant blinding; (6) instructor blinding; (7) assessor blinding; (8) more than 85% retention rate of at least one outcome; (9) intention-to-treat analysis; (10) between-group statistical comparison for at least one outcome; (11) point estimates and measures of variability provided for at least one outcome.

**Table 3:** Moderator analysis for the effects of physical activity vs. control intervention on global cognition.

Moderator	Level	Number of Trials	Sub-Analysis			Between-Group Homogeneity		
			SMD	95% CI	*I^2^* %	*Q*-Value	df(*Q*)	*p*-Value
Type	Aerobic exercise	8	0.60	0.32 to 0.88	43.6%	4.69	1	0.03
	Mixed exercises	10	0.24	0.06 to 0.41	0%			
Duration	≤12 weeks	8	0.39	0.15 to 0.63	0%	0.12	1	0.73
	≥13 weeks	10	0.45	0.20 to 0.69	50.3%			
	Low (≤2)	2	0.20	−0.17 to 0.58	0%	1.48	2	0.48
Frequency	Medium (3–4)	14	0.46	0.25 to 0.67	42.7%			
	High (5–7)	2	0.31	−0.13 to 0.76	0%			
Session time	≤45 min	7	0.66	0.33 to 0.99	45.4%	4.41	1	0.04
	>45 min	11	0.27	0.11 to 0.42	0%			
AD stage	Mild AD	7	0.20	−0.003 to 0.39	0%	6.26	2	0.04
	Mild to moderate AD	7	0.54	0.32 to 0.76	0%			
	Moderate to severe AD	4	0.75	0.03 to 1.47	79%			
Control type	Health education	1	0.63	0.06 to 1.20	0%	2.72	3	0.44
	Cognitive treatment	2	0.15	−0.33 to 0.63	0%			
	Social activity	5	0.66	0.11 to 1.21	72.7%			
	Usual medical treatment	11	0.35	0.19 to 0.52	0%			
Study quality	Good	13	0.36	0.21 to 0.51	66%	0.24	1	0.62
	Fair	5	0.51	0.05 to 1.06	2%			
	**Moderator**	**Number of Trials**	**β**	**95% CI**		***Q*-Value**	**df(*Q*)**	***p*-Value**
	Session time	18	−0.0105	−0.0197 to −0.0013		4.98	1	0.03
	Duration (weeks)	18	−0.0163	−0.0523 to 0.0196		0.79	1	0.37
	Frequency (per week)	18	0.0430	−0.1166 to 0.2026		0.28	1	0.60
	Total training time (during experiment)	18	−0.0001	−0.0002 to 0.0001		1.38	1	0.24
	Age	18	−0.0031	−0.03333 to 0.0271		0.042	1	0.84
	Male percentage	18	−0.0022	−0.0145 to 0.0101		0.12	1	0.73

AD = Alzheimer’s disease; CI = Confidence interval; df = degree of freedom; SMD = Standardized mean difference. ≤45 min = 30–45 min.

## Data Availability

The original research presented in the study is included in the article and Supplementary Materials. Further inquiries can be directed to the corresponding author.

## Supplementary Materials

The following supporting information can be downloaded at: https://www.mdpi.com/article/10.3390/ijerph19031216/s1, Figure S1: Distribution of true effects with prediction interval.
