# Supplementary material for: Physical Activity Improves Cognition and Activities of Daily Living in Adults with Alzheimer’s Disease: A Systematic Review and Meta-Analysis of Randomized Controlled Trials
Source: Int J Environ Res Public Health. 2022 Jan 22;19(3):1216. doi: 10.3390/ijerph19031216 (PMC8834999; doi:10.3390/ijerph19031216)
Supplement: Supplementary file 1 [file ijerph-19-01216-s001.zip › ijerph-1521207-supplementary.pdf]

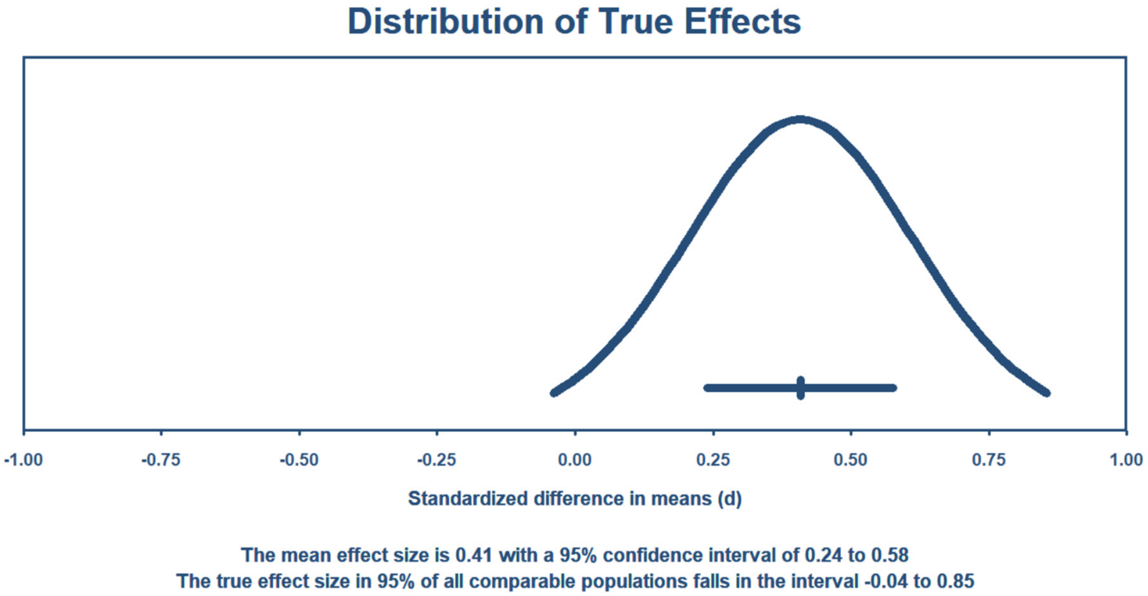

Figure S1. Distribution of true effects with prediction interval.
